# Acute Kidney Injury is Aggravated in Aged Mice by the Exacerbation of Proinflammatory Processes

**DOI:** 10.3389/fphar.2021.662020

**Published:** 2021-06-22

**Authors:** Laura Marquez-Exposito, Lucia Tejedor-Santamaria, Laura Santos-Sanchez, Floris A. Valentijn, Elena Cantero-Navarro, Sandra Rayego-Mateos, Raul R. Rodrigues-Diez, Antonio Tejera-Muñoz, Vanessa Marchant, Ana B. Sanz, Alberto Ortiz, Roel Goldschmeding, Marta Ruiz-Ortega

**Affiliations:** ^1^ Cellular Biology in Renal Diseases Laboratory, IIS-Fundación Jiménez Díaz, Universidad Autónoma Madrid, Madrid, Spain; ^2^ Red de Investigación Renal (REDinREN), Madrid, Spain; ^3^ Department of Pathology, University Medical Center Utrecht, Utrecht, Netherlands; ^4^ Division of Nephrology and Hypertension, IIS-Fundación Jiménez Díaz-Universidad Autónoma Madrid, Madrid, Spain

**Keywords:** aging, necroptosis, apoptosis, cellular senescence, inflammation, immunosenescence, klotho, acute kidney injury

## Abstract

Acute kidney injury (AKI) is more frequent in elderly patients. Mechanisms contributing to AKI (tubular cell death, inflammatory cell infiltration, impaired mitochondrial function, and prolonged cell-cycle arrest) have been linked to cellular senescence, a process implicated in regeneration failure and progression to fibrosis. However, the molecular and pathological basis of the age-related increase in AKI incidence is not completely understood. To explore these mechanisms, experimental AKI was induced by folic acid (FA) administration in young (3-months-old) and old (1-year-old) mice, and kidneys were evaluated in the early phase of AKI, at 48 h. Tubular damage score, KIM-1 expression, the recruitment of infiltrating immune cells (mainly neutrophils and macrophages) and proinflammatory gene expression were higher in AKI kidneys of old than of young mice. Tubular cell death in FA-AKI involves several pathways, such as regulated necrosis and apoptosis. Ferroptosis and necroptosis cell-death pathways were upregulated in old AKI kidneys. In contrast, caspase-3 activation was only found in young but not in old mice. Moreover, the antiapoptotic factor BCL-xL was significantly overexpressed in old, injured kidneys, suggesting an age-related apoptosis suppression. AKI kidneys displayed evidence of cellular senescence, such as increased levels of cyclin dependent kinase inhibitors p16ink4a and p21cip1, and of the DNA damage response marker γH2AX. Furthermore, p21cip1 mRNA expression and nuclear staining for p21cip1 and γH2AX were higher in old than in young FA-AKI mice, as well as the expression of senescence-associated secretory phenotype (SASP) components (*Il-6, Tgfb1*, *Ctgf,* and *Serpine1*). Interestingly, some infiltrating immune cells were p21 or γH2AX positive, suggesting that molecular senescence in the immune cells (“immunosenescence”) are involved in the increased severity of AKI in old mice. In contrast, expression of renal protective factors was dramatically downregulated in old AKI mice, including the antiaging factor Klotho and the mitochondrial biogenesis driver PGC-1α. In conclusion, aging resulted in more severe AKI after the exposure to toxic compounds. This increased toxicity may be related to magnification of proinflammatory-related pathways in older mice, including a switch to a proinflammatory cell death (necroptosis) instead of apoptosis, and overactivation of cellular senescence of resident renal cells and infiltrating inflammatory cells.

## Introduction

Acute kidney injury (AKI) is a common and devastating pathologic condition in part due its higher incidence in the elderly and its association with an increased short- and long-term mortality (Levey and James, 2017; Hounkpatin et al., 2019; Martin-Cleary et al., 2019; Logan et al., 2020). Moreover, AKI is closely related to chronic kidney disease (CKD) as AKI may accelerate CKD progression to end-stage renal disease (ESRD) and CKD predisposes to AKI (Venkatachalam et al., 2015; Siew et al., 2016; Ruiz-Ortega et al., 2020). All these facts underscore the importance of the research in this area. Furthermore, the cellular and molecular mechanisms of the increased sensitivity to AKI in elderly patients are incompletely understood (Mehran et al., 2019; Infante et al., 2020; Aleckovic-Halilovic et al., 2021), hampering the design of any preventive or therapeutic approaches.

Kidney tubular cells comprise the bulk of the kidney cell mass and may be injured by hypoxia, toxic compounds, metabolic disorders and proteinuria, among other factors. In response to an insult, tubular epithelial cells undergo phenotype changes associated with tubular function impairment and activation of inflammatory, fibrotic and cell death pathways, which may reflect a state of cellular senescence (Linkermann et al., 2014; Ruiz-Ortega et al., 2020). The initial phase of AKI is followed by a recovery phase characterized by activation of protective and regenerative mechanisms that restore epithelial properties and functions in surviving cells (Yang et al., 2010). Tubular cell death in AKI can involve several cell death pathways, such as apoptosis and regulated necrosis (Linkermann and Green, 2014). Cells dying by regulated necrosis release intracellular molecules, called damage-associated molecular patterns (DAMPs), which amplify the inflammatory response by the activation of neutrophils and other immune cells in a process termed necroinflammation. There are several forms of regulated necrosis, including necroptosis, ferroptosis, and pyroptosis (Newton and Manning, 2016). Necroptosis, the best-characterized form of regulated apoptosis, is elicited by the binding of the receptor-interacting protein 1 (RIPK1) to RIPK3, leading to its oligomerization and autophosphorylation. Then, the active RIPK1-RIPK3 complex (also called necrosome) activates the pseudokinase mixed lineage kinase domain-like protein (MLKL), which translocates to the cellular membrane, causing cell membrane permeabilization, rupture, and subsequent cell death (Newton and Manning, 2016). Necroptosis plays an important role in experimental AKI, as described in renal ischemia/reperfusion injury (IRI), folic acid (FA)-AKI and cisplatin nephropathy (Linkermann et al., 2013; Linkermann and Green, 2014; Xu et al., 2015; Martin-Sanchez et al., 2017; Martin-Sanchez et al.,2018a). Ferroptosis, a caspase-independent cell death pathway, is characterized by reduced glutathione activity or content, reduced glutathione peroxidase 4 (GPX4) protein levels, massive lipid peroxidation and cell loss (Martin-Sanchez et al., 2020). Targeting ferroptosis by chemical inhibition or gene expression modulation reduced tubular injury and improved renal function in different experimental models, including IRI and FA-AKI (Martin-Sanchez et al., 2017; Martin-Sanchez et al., 2020).

Cellular senescence represents a maladaptive response to AKI, characterized by prolonged cell-cycle arrest (Melk et al., 2004; Bonventre, 2014; Gorgoulis et al., 2019). Following an initial insult, DNA damage activates a protective mechanism consisting in the arrest of the cell cycle and the activation of the DNA damage response (DDR) to facilitate DNA repair. After successful DNA repair, cells re-enter the cell cycle (Branzei and Foiani, 2008). Nevertheless, persistent activation of this protective mechanism can contribute to damage, as observed in disease conditions associated with cellular senescence (Gire and Dulic, 2015). Regarding the kidney, accumulation of senescent (in particular tubular epithelial) cells has been implicated in regeneration failure and AKI-to-CKD transition (Schmitt and Cantley, 2008; O’Sullivan et al., 2017; Kim et al., 2019). In this sense, prolonged tubular epithelial cell-cycle arrest, sustained inflammation, and impaired mitochondrial function can contribute to CKD progression (Levey and James, 2017; Andrade et al., 2018; Sato and Yanagita, 2018; Jiang et al., 2020). Cellular senescence or premature aging in the kidney is characterized by increased expression of some cell-cycle-related molecules such as the cyclin kinase inhibitors p16ink4a, p21cip, and p53 (Melk et al., 2004; Andrade et al., 2018; Knoppert et al., 2019; Koyano et al., 2019). Senescent cells are also characterized by a detrimental secretome known as senescence-associated secretory phenotype (SASP) (Melk et al., 2004; Bonventre, 2014). This secretome is enriched with pro-inflammatory cytokines, growth factors and profibrotic proteins such as IL-6, TGF-β, CTGF/CCN2 and PAI-1 (Acosta et al., 2013; Zhou et al., 2020) and is able to spread the senescence phenotype to neighboring cells (paracrine senescence) (Acosta et al., 2013), and to promote kidney fibrosis (Ferlicot et al., 2003; Melk et al., 2004; McGlynn et al., 2009; Yang et al., 2010; Günther et al., 2017; Valentijn et al., 2018). While inflammation is one of the first steps in tissue repair, persistent inflammation contributes to CKD progression (Cao et al., 2015; Rabb et al., 2016; Sato and Yanagita, 2018). Cytokines and interleukins within the SASP contribute to enduring inflammation and to further tubular cell injury and dysfunction (Kirkland and Tchkonia, 2017). Moreover, a low-grade inflammatory milieu is known to be present in the aged tissues, a condition named “inflammaging” (Greene and Loeser, 2015; Rea et al., 2018). “Immunosenescence” is a related concept, in which the dysfunctional immune response in the elderly presents characteristics related to cellular senescence and promotes inflammation, thus playing a crucial role in inflammaging (Sato and Yanagita, 2019; Schroth et al., 2020).

In the present study, we sought to elucidate the potential cellular and molecular mechanisms contributing to the increased severity of AKI in old age. To this aim, we have investigated whether aging-related processes, such as induction of cellular senescence, inflammaging and loss of renal protective factors, can modulate tubular damage, including cell death pathways activation and phenotype changes induced by AKI. Previous experimental studies have reported age-related exacerbation of renal injury in different AKI models. Now, we have investigated the FA-AKI model that presents a different mechanism of kidney injury (crystalluria with intratubular obstruction) than those in prior studies (cytokine storm) or on exogenous (cisplatin) or endogenous (heme) molecules that are directly toxic to tubular cells (Maddens et al., 2012; Nath et al., 2013; Wen et al., 2015). Aging is a process that has no fixed start date and does not occur suddenly. Rather, human glomerular filtration rate starts decreasing progressively from age 18–24 years (Wetzels et al., 2007). Most previous experimental studies on AKI and aging used mice from 15 to 18 months old (Maddens et al., 2012; Nath et al., 2013; Wen et al., 2015). However, in a sepsis AKI model an increase in mortality was already observed at 12 months (Maddens et al., 2012). Therefore, this time point was chosen to investigate early age-associated changes that could be responsible for increased AKI susceptibility.

## Materials and Methods

### Experimental Model of Acute Kidney Injury Induced by Toxins

C57BL/6 mice were originally obtained from JAX™ Mice (Charles River Europe laboratory) and then the mouse colony breeding was maintained in the Fundación Jimenez Diaz Animal facilities, following JAX™ recommendations (Jackson Laboratory, 2007). Animals were fed with a standard diet provided by the animal facilities. Young (3-month-old) and old (1-year-old) C57BL/6 male mice were injected intraperitoneally with 125 mg/kg folic acid (FA) dissolved in sodium bicarbonate. Body weight was similar in young and old mice (26.1 g in young vs. 29.8 g in older mice). Previous studies have demonstrated that the lethal dose for FA (lethality dependent on AKI) varies by more than 3-fold in different mouse strains (Parchure et al., 1985). In addition, in a sepsis AKI model has reported lethality at 12 months (Maddens et al., 2012). Therefore, to decrease the high risk of death in old mice, we used a lower FA dose than in prior studies (125 mg/kg instead of 250 mg/kg) (Linkermann et al., 2013; Martin-Sanchez et al., 2018a), and mice of 12 months. As observed in results, this dose induced a significant tubular damage with no death associated at this time point. Five to ten mice per group were studied in the early phase of AKI, after 48 h of FA injection. Untreated mice of the same age were used as their corresponding controls.

Animals were euthanatized by CO_2_ inhalation. The kidneys were perfused *in situ* with saline before removal, and half of each kidney (2/4) was fixed, embedded in paraffin, and used for immunohistochemistry, while the rest was snap-frozen in liquid nitrogen for renal cortex RNA and protein studies. Kidneys from all groups were compared to control kidneys from young mice, expressing results as fold-change over control values of 1.

### Protein Studies

Total proteins were isolated from frozen kidney tissue using an appropriate lysis buffer as previously described (Rodrigues-Diez et al., 2013) and quantified using a BCA protein assay kit (ThermoScientific). Proteins (50 μg) were separated on 8–15% acrylamide gels using the SDS-PAGE, as described (Rodrigues-Diez et al., 2013). Briefly, after electrophoresis, samples were transferred on to polyvinylidenedifluoride membranes (Millipore) blocked in TBS containing 0.1% Tween 20 (TBST) and 5% dry non-fat milk for 1 h at room temperature and incubated in the same buffer with different primary antibodies overnight at 4°C. After washing with TBST, membranes were incubated with the appropriate HRP (horseradish peroxidase)-conjugated secondary antibody (Invitrogen) 1 h at room temperature and developed using an ECL kit (Amersham Biosciences). Results were analyzed by LAS 4,000 and Amersham Imager 600 (GEHealthcare) and densitometered by Quantity One software (Biorad). The following primary antibodies were employed [dilution]: MLKL ([1:1,000], ab172868, abcam), α-tubulin ([1:5,000], T5168, Sigma-Aldrich) and α-Cleaved Caspase 3 ([1:1,000, #9661S, Cell Signaling). The evaluation of IL-6 in kidney tissue was done by ELISA (BD Biosciences, Cat. No. 555240) following the instructions provided by the manufacturer.

### Histology and Immunohistochemistry

Paraffin-embedded kidney sections were stained using standard histology procedures, as described elsewhere (Rodrigues-Diez et al., 2013). Tubular damage and inflammatory infiltrate were scored as arbitrary units on periodic acid-Schiff (PAS, Sigma-Aldrich) stained slides as previously described (Zoja et al., 2002). Immunostaining was carried out in 3 μm thick tissue sections. Antigens were retrieved using the PTlink system (DAKO) with sodium citrate buffer (10 mM) adjusted to pH 6–9, depending on the immunohistochemical marker. Endogenous peroxidase was blocked. Tissue sections were incubated for 1 h at room temperature with 1X Casein Solution (Vector Laboratories) to eliminate non-specific protein binding sites. Primary antibodies were incubated overnight at 4°C and diluted in antibody solution (DAKO). Specific HRP-conjugated (DAKO) or biotinylated secondary antibodies (Amersham Biosciences) were used for 1 h followed by Avidin-Biotin Complex incubation (Vector Laboratories). Signal was developed with substrate solution and 3,3-diaminobenzidine as a chromogen (Abcam). Then sections were counterstained with Carazzi’s haematoxylin (Richard Allan Scientific). The primary antibodies used were [dilution]: KIM-1 ([1:500]; AF 1817, R&D), P21 ([1:2,000, Ab188224, Abcam), γH2AX [1:1,000], NB1002280 Novus Biological), BCL-xL ([1:4,000], ab178844, Abcam), F4/80 ([1:50]; MCA497, Bio-Rad), CD3 ([1:100], A0452, DAKO), Myeloperoxidase ([1X], IS511, DAKO) and 4-Hydroxynonenal ([1:1,000], Ab46545, Abcam). Specificity was checked by omission of primary antibodies (not shown). Quantification was made by using the Image-Pro Plus software (Maryland, United States) determining the positive staining area relative to the total area or counting positive staining manually (in the case of inflammatory cells), in 5–10 randomly chosen fields (× 200 magnification).

### Gene Expression Studies

RNA from renal cortex was isolated with TriPure reagent (Roche). cDNA was synthesized by a High Capacity cDNA Archive kit (Applied Biosystems) using 2 μg of total RNA and following the manufacturer’s instructions. Quantitative gene expression analysis was performed on an AB7500 fast real-time PCR system (Applied Biosystems) using fluorogenic TaqMan MGB probes and primers designed by Assay-on-Demand™ gene expression products. Mouse assays IDs were: *p21cip1*: Mm00432448_m, *p16ink4a*: Mm00494449_m1, *Klotho*: Mm00502002_m1, *Bcl2l1* Mm004337783_m1, *Il6* Mm00446190_m1, *Lcn2* Mm01324470_m1, *Havcr1 o Kim1*: Mm00506686_m1, *Ctgf/Ccn2:* Mm01192933_g1, *Ccl-2*: Mm00441242_m1, *Ppargc1a*: Mm01208835_m1, *Tgfβ1*: Mm01178820, *Mlkl:* Mm01244219_m1, *Ripk3*: Mm00444947_m1, *Serpine1*: Mm00435858_m1, *Ccl5*: Mm01302428_m1, *Cxcl1*: Mm04207460_m1, *Cxcl2*: Mm00436450_m1, *Cxcl5*: Mm00436451_m1, *Cxcl10*: Mm00445235_m1, and *Gpx4*: Mm00515041_m1. Data were normalized to *Gapdh*: Mm99999915_g1. The mRNA copy numbers were calculated for each sample by the instrument software using Ct value (“arithmetic fit point analysis for the lightcycler”). Results were expressed in n-fold, calculated relative to young mice control group after normalization against *Gapdh*.

### Statistical Analysis

Results are expressed as n-fold increase with respect to the average of young control mice as mean ± standard deviation of the mean (±SD). The Shapiro-Wilk test was used to evaluate sample Normality distribution. If the samples followed the Gaussian distribution, a one-way ANOVA followed by the corresponding *post-hoc* analyses, were used. To compare non-parametric samples, a Kruskal-Wallis and a subsequent *post-hoc* analysis was performed. Statistical analysis was conducted using GraphPad Prism 8.0 (GrahPad Software, San Diego California United States). Values of *p* < 0.05 were considered statistically significant.

## Results

### Experimental AKI Induced by Folic Acid Administration is Characterized by More Severe Tubular Injury and Inflammatory Cell Infiltration in Old Mice.

**FIGURE 1:**
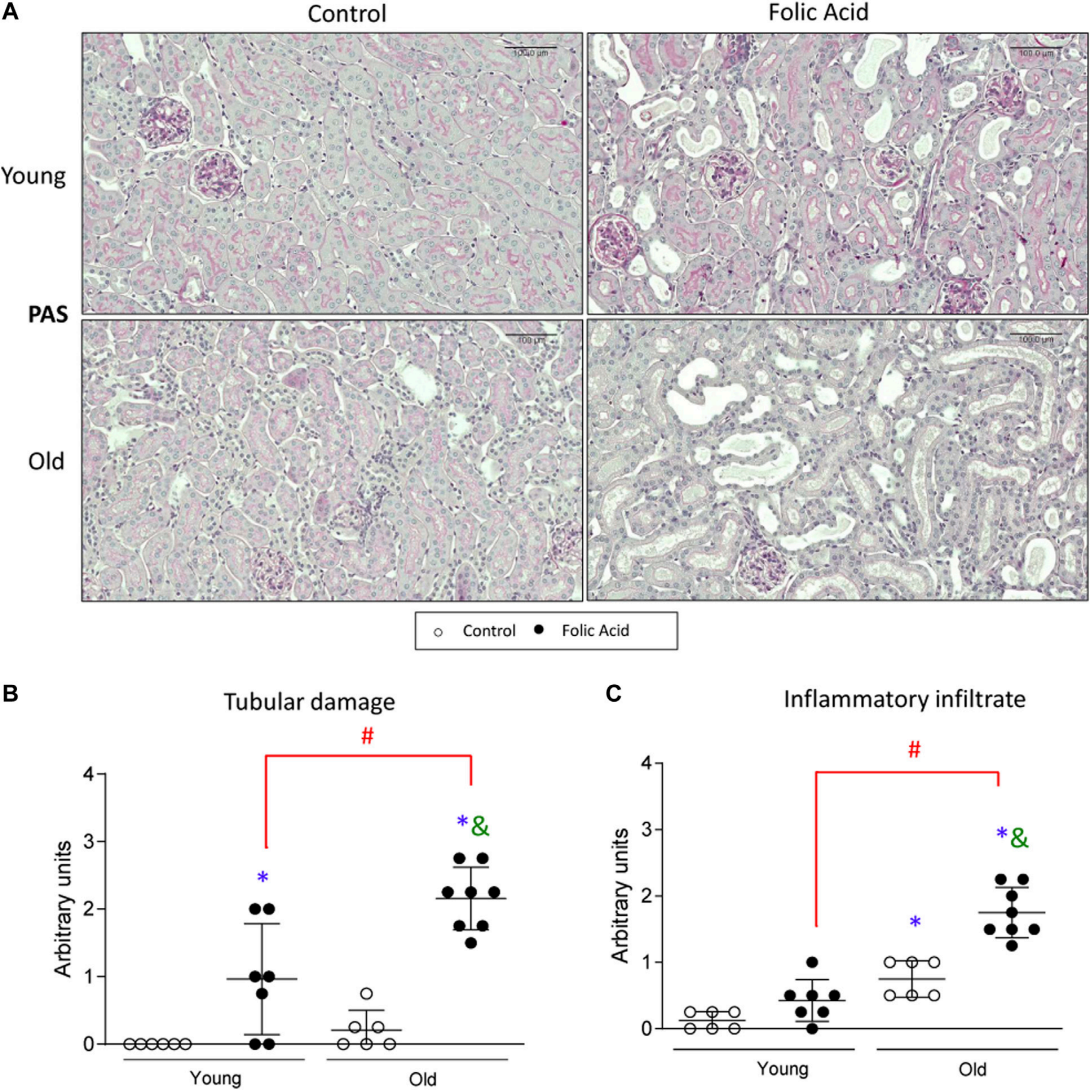
Histological characterization of renal lesions in the acute phase of the folic acid nephropathy in young and old mice. Folic Acid (FA; 125 mg/kg) was injected in 3-months-old (Young) and 1-year-old (Old) C57BL/6 mice and kidneys were studied after 48 h. The morphological lesions were evaluated by Periodic Acid-Schiff stained kidney sections. **(A)** Figure shows representative micrographs from each group and the quantification, from 0 to 4, of **(B)** tubular damage and **(C)** inflammatory infiltrate. Scale bars = 100 μm. Data are shown as arbitrary units and expressed as mean ± SD of *n* = 6–8 animals per group. *p < 0.05 vs. control young mice, ^#^ *p* < 0.05 vs. FA-injected young mice, ^&^ *p* < 0.05 vs. control old mice. The non-parametric Kruskal-Wallis statistical test was performed.

Kidney injury was studied 48 h after the injection of a low dose (125 mg/kg) of FA to young (3-months-old) and old (1-year-old) mice. The morphological changes showed that FA administration induced tubular injury was more severe in old than in young mice (Figures 1A,B). Moreover, in FA-treated mice the recruitment of inflammatory cells in the kidney was higher in old than in young mice (Figures 1A,C).

**FIGURE 2:**
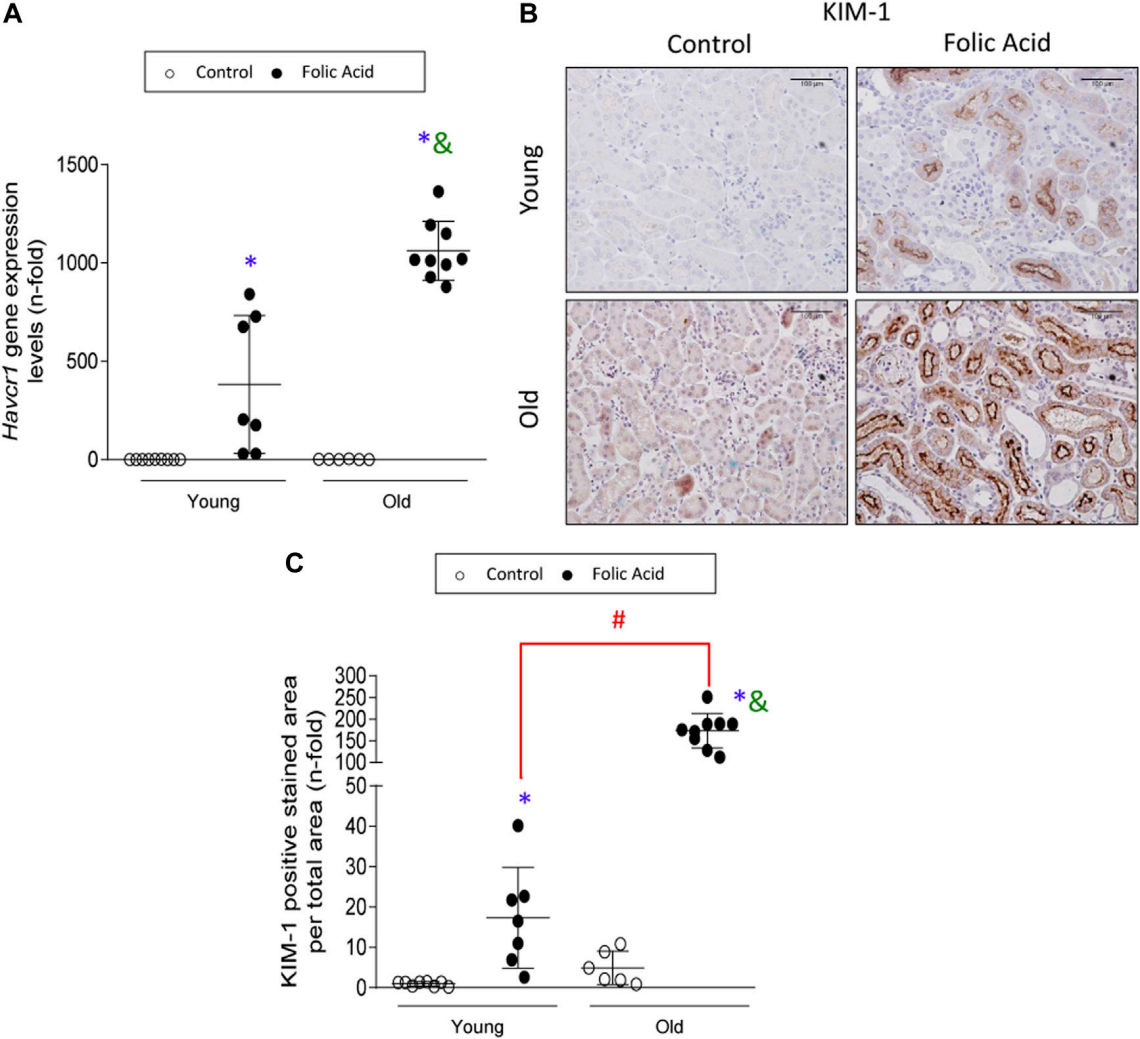
The damage biomarker KIM-1 is overexpressed in injured tubules of old mice. Folic Acid (FA; 125 mg/kg) was injected in 3-months-old (Young) and 1-year-old (Old) C57BL/6 mice and kidneys were studied after 48 h. **(A)** Total mRNA was isolated from frozen sections of whole kidneys and qRT-PCR was performed to determine gene expression levels of *Havcr-1*. **(B)** Representative microphotographs of KIM-1 expression levels evaluated by immunohistochemistry and **(C)** its quantification of stained area per total area. Scale bars = 100 μm. Data are shown as n-fold and expressed as mean ± SD of *n* = 6–9 animals per group. *p < 0.05 vs. control young mice, ^#^ *p* < 0.05 vs. FA-injected young mice, ^&^ *p* < 0.05 vs. control old mice. The non-parametric Kruskal-Wallis statistical test was performed.

Tubular damage was further evaluated at molecular levels by studying the gene expression levels of the tubular injury biomarker *Havcr-1*, that encodes the KIM-1 protein (Beker et al., 2018; Griffin et al., 2019; Gohda et al., 2020). Havcr-1/KIM-1 is an early marker of kidney injury in rodent AKI induced by IRI or nephrotoxic drugs (Bignon et al., 1976; Amin et al., 2004; Prozialeck et al., 2007). Kidney *Havcr-1* gene expression was increased in FA-induced AKI in both young and old mice (Figure 2A). In control young kidneys, KIM-1 protein expression was minimal (Figures 2B,C). However, in FA-injured kidneys apical KIM-1 staining was observed (Figure 2B), in accordance with previous studies (Han et al., 2002). Importantly, the quantification of KIM-1 staining showed dramatically higher tubular KIM-1 protein expression levels in old than in young injured kidneys (Figure 2C).

**FIGURE 3:**
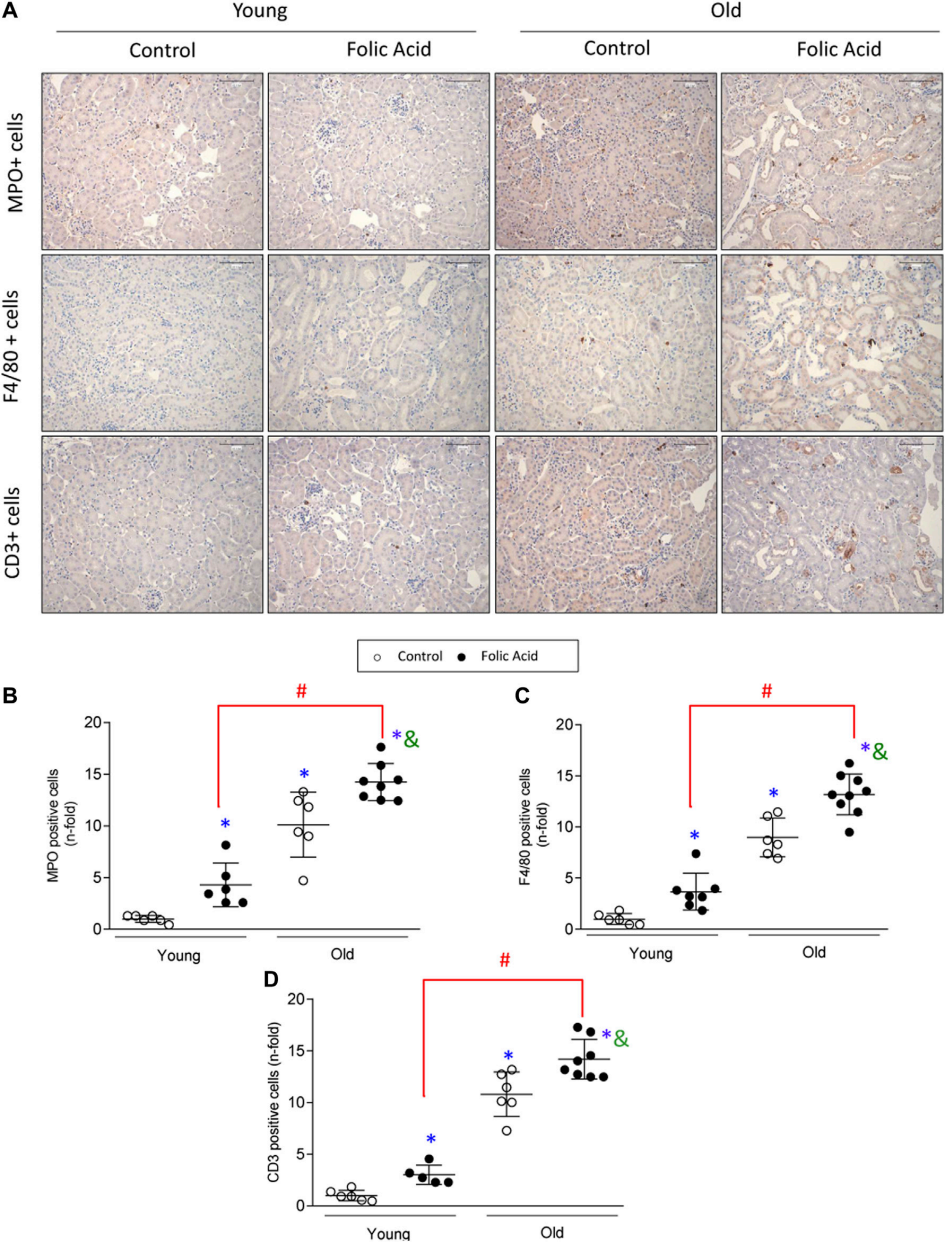
Characterization of inflammatory infiltrate in the acute phase of folic acid (FA) nephropathy in young and old mice. Folic Acid (FA; 125 mg/kg) was injected in 3-months-old (Young) and 1-year-old (Old) C57BL/6 mice and kidneys were studied after 48 h. Inflammatory cell infiltration was evaluated using antibodies against myeloperoxidase (neutrophils), F4/80 (monocytes/macrophages/dendritic cells) and CD3 (T lymphocytes). **(A)** Representative micrographs from each group. Scale bars = 100 μm. **(B–D)** Quantification of MPO **(B)**, F4/80 **(C)**, and CD3 **(D)** positive cells. Data are shown as n-fold and expressed as mean ± SD of *n* = 6–9 animals per group. *p < 0.05 vs. control young mice, ^#^ *p* < 0.05 vs. FA-injected young mice, ^&^ *p* < 0.05 vs. control old mice. The parametric one-way ANOVA statistical test was performed.

**FIGURE 4:**
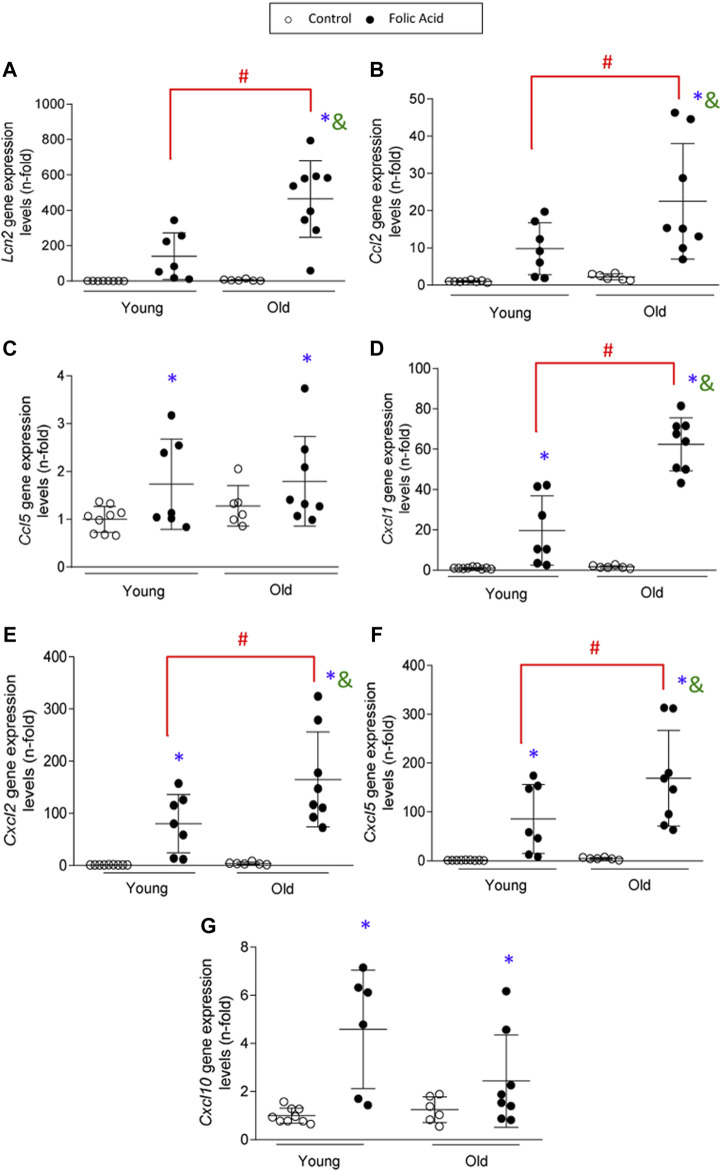
Kidney expression of proinflammatory genes in the acute phase of folic acid (FA) nephropathy in young and old mice. Folic Acid (FA; 125 mg/kg) was injected in 3-months-old (Young) and 1-year-old (Old) C57BL/6 mice. Kidneys were studied after 48 h and qRT-PCR was performed to assess *Lcn2* **(A)**, *Ccl2* **(B)**, *Ccl5* **(C)**, *Cxcl1* **(D)**, *Cxcl2* **(E)**, *Cxcl5* **(F)**, and *Cxcl10* **(G)** gene expression levels. Data are shown as n-fold and expressed as mean ± SD of *n* = 6–9 animals per group. *p < 0.05 vs. control young mice, ^#^ *p* < 0.05 vs. FA-injected young mice, ^&^ *p* < 0.05 vs. control old mice. The parametric one-way ANOVA statistical test was performed.

To further characterize the kidney infiltrating cells, immunohistochemistry was done using specific markers of neutrophils (Mieloperoxidase), macrophages (F4/80 + cells) and T-lymphocytes (CD3+ cells) (Figure 3A). Infiltration by neutrophils, monocytes/macrophages and CD3+ T cells was significantly higher in old than in young mice with FA-induced AKI (Figures 3B–D). Next, changes in gene expression of key inflammatory markers were evaluated by qRT-PCR in mouse kidneys. There were no differences in gene expression levels of proinflammatory factors between young and old control mice. In contrast, all of them were increased in response to FA administration both in young and old mice compared to untreated mice (Figure 4). The proinflammatory marker *Lcn2,* which encodes the kidney damage biomarker N-GAL (Wang et al., 2007), and the chemokine *Cxcl1*, which plays a key role in neutrophil recruitment (Chung and Lan, 2011), were significantly upregulated in FA kidneys from old compared to AKI young ones (Figure 4). In addition, other cytokines and chemokines, such as *Ccl2* and *Cxcl2* were also higher in old vs. young FA kidneys, but no differences were found in the case of *Ccl5* and *Cxcl10* (Figure 4).

### Ferroptosis is Increased in Old Folic Acid-Induced Acute Kidney Injury When Compared to Young Folic Acid Kidneys

**FIGURE 5:**
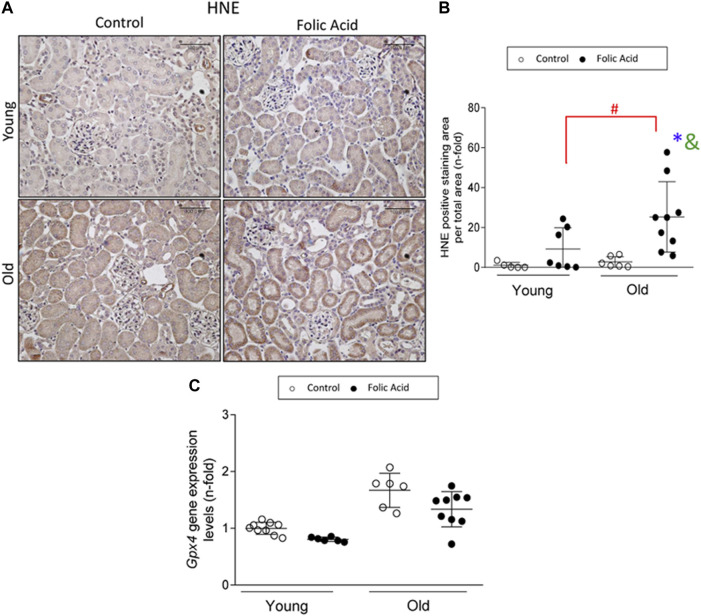
Ferroptosis death pathway is significantly increased in FA-induced AKI in old vs. young mice. Folic Acid (FA; 125 mg/kg) was injected in 3-months-old (Young) and 1-year-old (Old) C57BL/6 and kidneys were studied after 48 h **(A, B)** 4-Hidroxynonenal (HNE) immunohistochemistry was performed. **(A)** Representative pictures from each group and **(B)** the quantification of stained area per total area was performed. **(C)** Renal gene expression levels of *Gpx4* were studied by qRT-PCR. Scale bars = 100 μm. Data are expressed as mean ± SD of *n* = 6–9 animals per group. *p < 0,05 vs. Young mice control. The non-parametric Kruskal-Wallis statistical test was performed.

Ferroptosis is a regulated death pathway involved in the first wave of death in FA-AKI (Martin-Sanchez et al., 2017). To determine if ferroptosis was overactivated in response to FA administration in old mice, lipid peroxidation, a final ferroptosis target, was evaluated by HNE immunohistochemistry. An increase in HNE staining was found in FA-injected kidneys from old mice compared to young ones (Figure 5A). GPX4 reduction was previously described in FA-AKI (Martin-Sanchez et al., 2017). However, gene expression levels of *Gpx4* were not diminished in young FA-kidneys at the low dose used in the present study, whereas in old mice there was a slight, but not significant, diminution of *Gpx4* mRNA levels (Figure 5B).

### Necroptosis Components are Overexpressed in Response to Folic Acid Administration in Old Mice.

**FIGURE 6:**
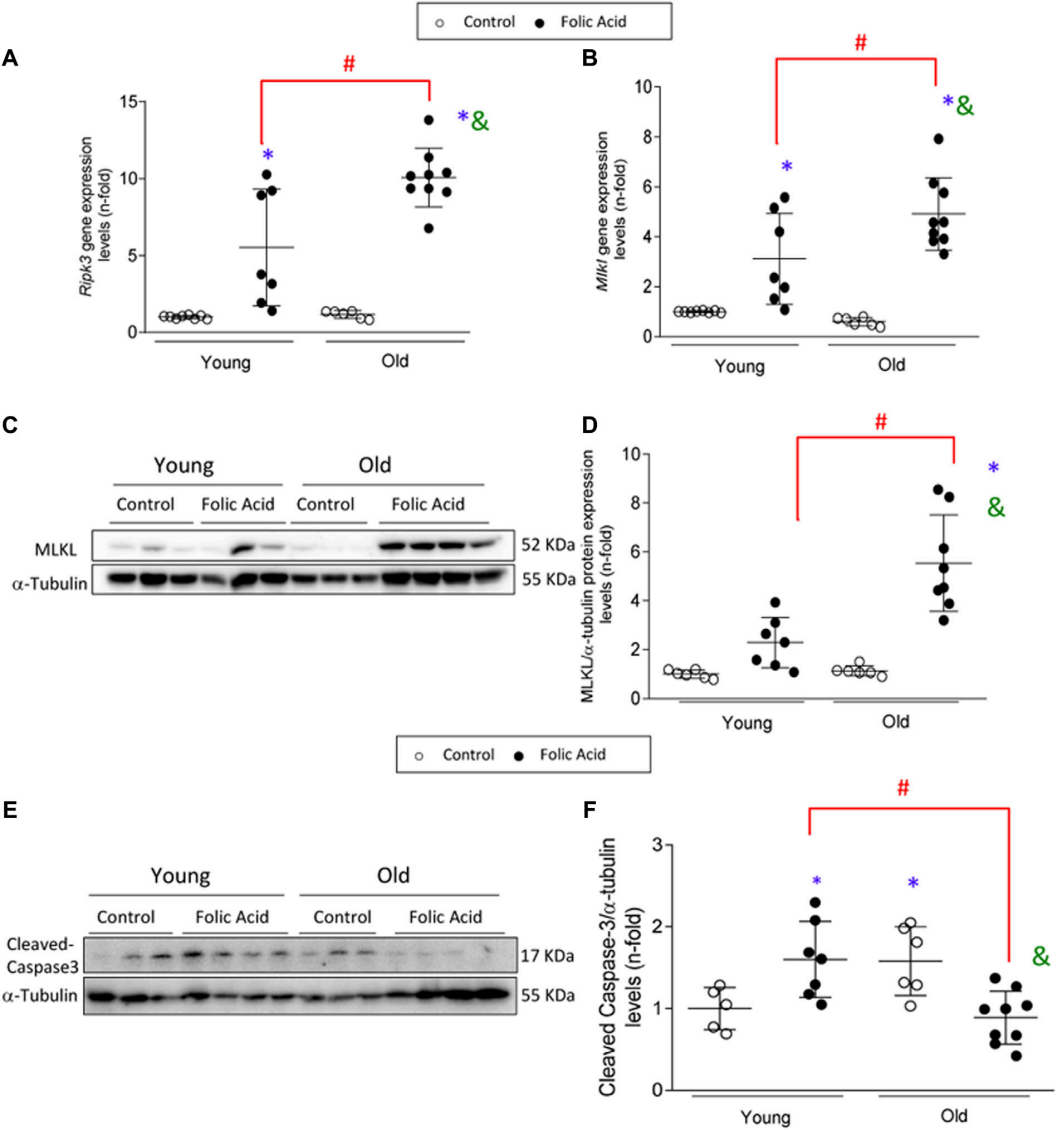
Upregulation of the necroptosis pathway in the acute phase of folic acid (FA) nephropathy in old and young mice. Folic Acid (FA; 125 mg/kg) was injected in 3-months-old (Young) and 1-year-old (Old) C57BL/6 mice and kidneys were studied after 48 h. The renal gene expression levels of *Ripk3* **(A)** and *mlkl* **(B)** were evaluated by qRT-PCR. **(C–F)** MLKL protein and active caspase-3 (represented by cleaved caspase-3) were determined by Western blot. α-tubulin was used as loading control. **(C, E)** Representative blots and **(D, F)** their quantification. Data are shown as n-fold and expressed as mean ± SD of *n* = 5–9 animals per group. *p < 0.05 vs. control young mice, ^#^ *p* < 0.05 vs. FA-injected young mice, ^&^ *p* < 0.05 vs. control old mice. The parametric one-way ANOVA statistical test was performed.

Necroptosis is a cell death pathway associated with inflammation (Newton and Manning, 2016). The renal expression of the main components of the necroptosis pathway was evaluated in the AKI model. Renal *Ripk3* and *Mlkl* gene expression levels were increased in FA-injected mice compared to controls, as previously described (Figures 6A,B) (Martin-Sanchez et al., 2017; Martin-Sanchez et al., 2018a). These increases were markedly higher in old mice showing a significantly higher gene expression of both markers (Figure 6A), as well as MLKL total protein expression (Figures 6C,D).

### Apoptosis is not Involved in the Folic Acid-Acute Kidney Injury severity Observed in Old Mice Compared to Young Mice.

Apoptosis is also involved in FA-AKI (Justo et al., 2006). As previously described, activation of caspase 3 was found during AKI in young mice, as evidenced by increased levels of mature caspase 3 (Justo et al., 2006). However, this was not the case for old mice with AKI, in whom active caspase 3 was not increased (Figures 6E,F). These data would suggest a possible switch of AKI-induced cell death pathway from a non-inflammatory apoptotic cell death to a proinflammatory necroptosis cell death in old age.

### Molecular Senescence is Activated in Acute Kidney Injury and Exacerbated in Old Mice

**FIGURE 7:**
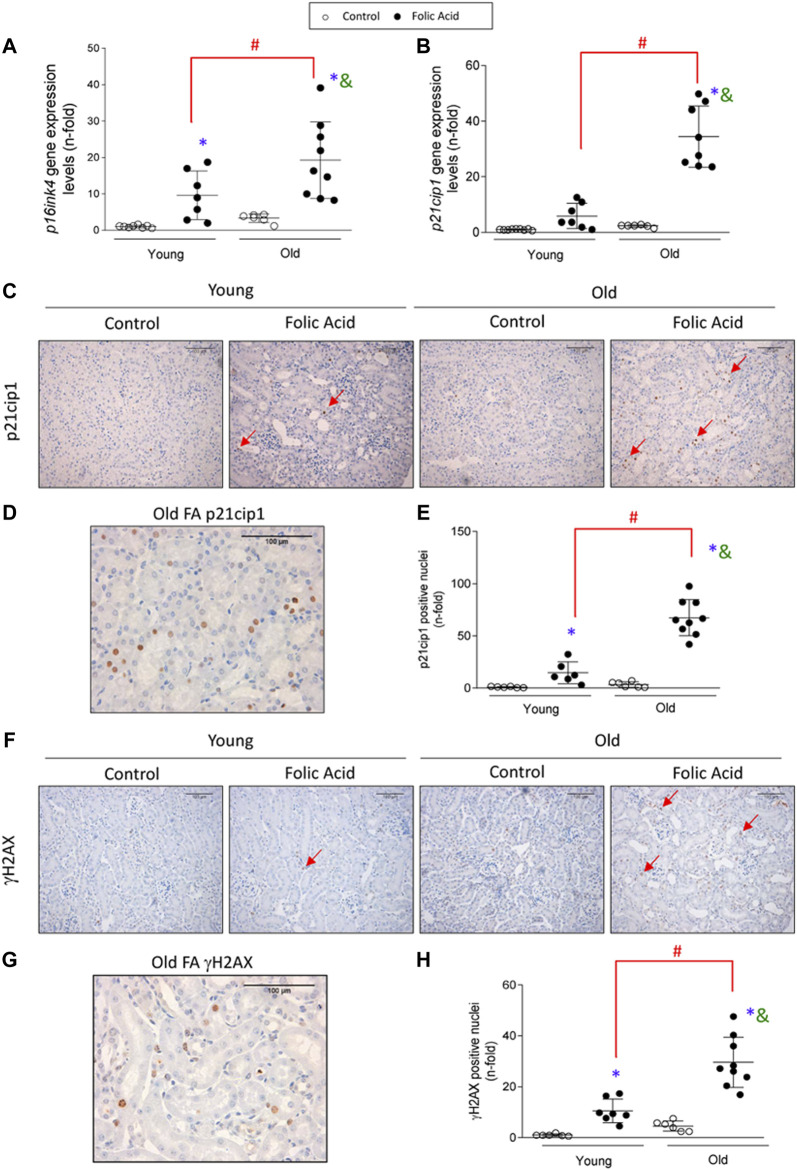
Kidney expression of cell-cycle arrest and DDR markers pathway in the acute phase of folic acid (FA) nephropathy in old and young mice. Folic Acid (FA; 125 mg/kg) was injected in 3-months-old (Young) and 1-year-old (Old) C57BL/6 mice and kidneys were studied after 48 h **(A, B)** Kidney *p16ink4a* **(A)** and *p21cip1* **(B)** gene expression levels. **(C–E)** p21cip1 immunohistochemistry was conducted. **(C)** Representative microphotographs of p21cip1 showing nuclear staining and **(D)** detail of a FA-injured old mouse kidney showing positive nuclear p21cip1 staining. **(E)** Nuclear p21cip1 quantification. **(F–H)** γH2AX immunohistochemistry was performed. **(F)** Representative micrographs of γH2AX and **(G)** detail of a FA-injured old mouse kidney showing positive nuclear γH2AX staining. **(H)** Nuclear γH2AX quantification. Red arrows indicate positive nuclear staining. Scale Bars = 100 μm. Data are expressed as mean ± SD of *n* = 6–9 animals per group. *p < 0.05 vs. control young mice, ^#^ *p* < 0.05 vs. FA-injected young mice, ^&^ *p* < 0.05 vs. control old mice. The parametric one-way ANOVA statistical test was performed, except for γH2AX quantification, in which a non-parametric Kruskal-Wallis statistical test was conducted.

Cellular senescence was induced in the early phase of FA-AKI, as observed by increased gene expression levels of the cyclin dependent kinase inhibitors *p16ink4a* and *p21cip1* at 48 h (Figures 7A,B). Importantly, *p16ink4a* and *p21cip1* upregulation was exacerbated by aging, evidenced by higher expression in old than in young FA-injured kidneys (Figures 7A,B). Moreover, nuclear p21cip1 immunostaining was observed in FA-injected mice (Figures 7C,D) and the number of p21cip1 positive nuclei was significantly higher in old than in young FA-injured kidneys (Figure 7E). To further analyze senescent-related mechanisms, the DNA damage response marker γH2AX was evaluated. In response to FA administration, nuclear γH2AX expression was also increased in both young and old mice and showed a higher upregulation in old mice (Figures 7F–H). Interestingly, some infiltrating immune cells were also p21cip1 or γH2AX positive (Figures 7C,D,F,G), suggesting that immunosenescence and inflammaging are involved in the aggravated AKI response to FA in old mice.

**FIGURE 8:**
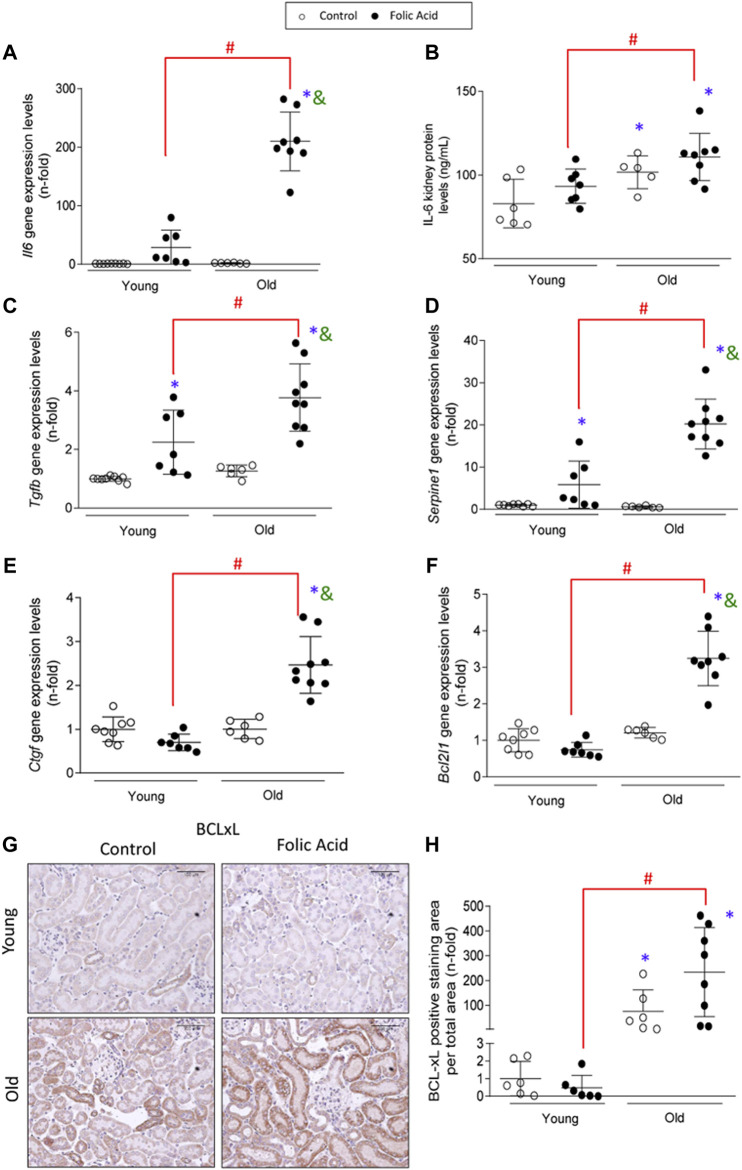
Increased expression of senescence-associated secretory phenotype components (SASP) and anti-apoptotic proteins in old injured kidneys. Folic Acid (FA; 125 mg/kg) was injected in 3-months-old (Young) and 1-year-old (Old) C57BL/6 mice and kidneys were studied after 48 h. Gene expression levels of *Il6* **(A)**, *Tgfβ* **(C)**, *Serpine1* **(D)**, and *Ctgf* **(E)** were determined by qRT-PCR. **(B)** Total protein of renal extracts of IL-6 were evaluated by ELISA. **(F)** Gene expression levels of *Bcl2l1* were evaluated by qRT-PCR. **(G, H)** BCL-xL protein was evalated by immunohistochemistry. **(G)** Representative microphotographs of BCL-xL and **(H)** its quantification of stained area per total area. Data are shown as n-fold and expressed as mean ± SD of *n* = 6–9 animals per group. *p < 0.05 vs. control young mice, ^#^ *p* < 0.05 vs. FA-injected young mice, ^&^ *p* < 0.05 vs. control old mice. The parametric one-way ANOVA statistical test was performed, except for the BCL-xL quantification, in which a non-parametric Kruskal-Wallis statistical test was conducted.

Another feature of senescent cells is the increased production of SASP. The analysis of the gene expression levels of SASP components *Tgfβ1*, *Il6*, *Ctgf/Ccn2* and *Serpine1* (which encodes PAI-1) and the IL-6 protein levels assayed by ELISA, in the early phase of AKI, showed that all of the evaluated SASP components were higher in old AKI mice compared to the young ones (Figures 8A–E).

Senescent cells are protected from apoptosis (Knoppert et al., 2019). Here, the antiapoptotic factor BCL-xL, an important B-cell lymphoma 2 (BCL-2) family member central to senescent cell apoptosis resistance (Chang et al., 2016; Yosef et al., 2016) was evaluated at gene (*Bcl2l1*) and protein (BCL-xL) levels. Both *Bcl2l1* gene and BCL-xL protein expression levels were upregulated in injured kidneys of old mice compared to the young ones (Figures 8F–H). Interestingly, overexpression of BCL-xL was observed in old control mice compared to young mice (Figures 8G,H).

### Age-Related Loss of Protective Factors

**FIGURE 9:**
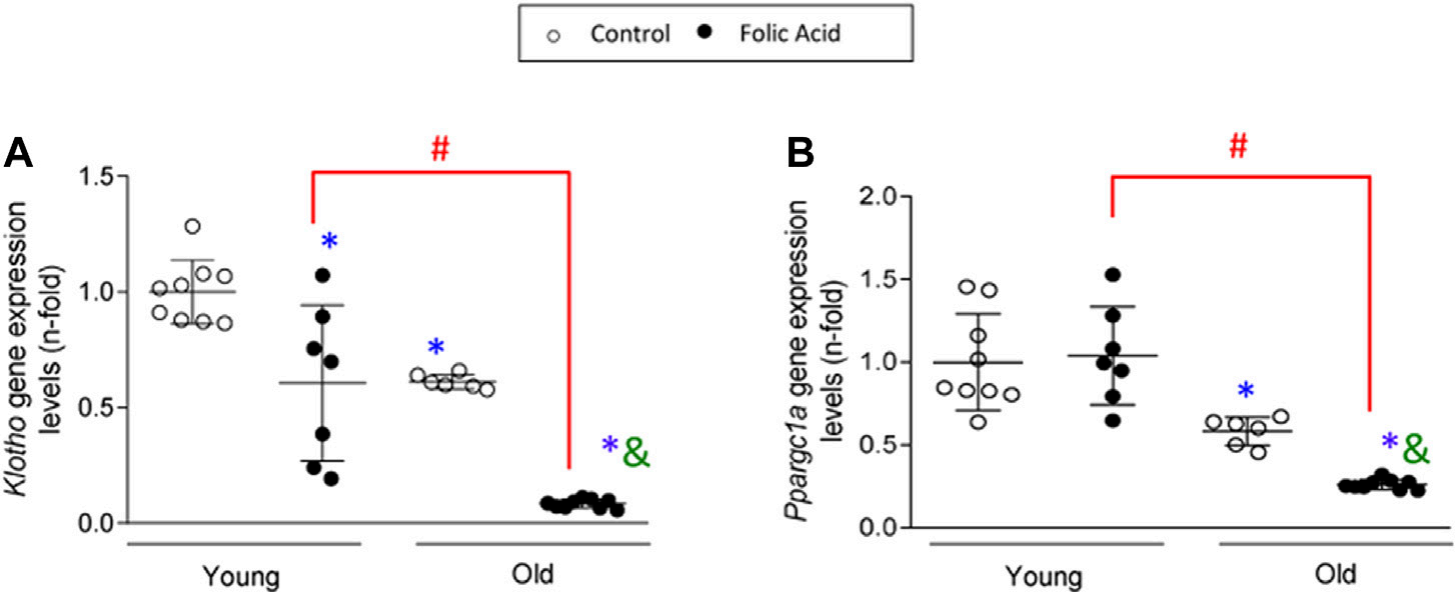
Loss of protective factors in old kidneys is exacerbated in response to folic-acid (FA) administration. Folic Acid (FA; 125 mg/kg) was injected in 3-months-old (Young) and 1-year-old (Old) C57BL/6 mice and kidneys were studied after 48 h. Kidney *Klotho* **(A)** and *Ppargc1a* **(B)** gene expression levels were assessed by qRT-PCR. Data are shown as n-fold and expressed as mean ± SD of *n* = 6-9 animals per group. *p < 0.05 vs. control young mice, ^#^ *p* < 0.05 vs. FA-injected young mice, ^&^ *p* < 0.05 vs. control old mice. The parametric one-way ANOVA statistical test was performed.

Klotho is an anti-aging protein of kidney origin that is lost very early in the course of AKI or CKD (Moreno et al., 2011; Fernandez-Fernandez et al., 2018; Sanchez-Niño et al., 2019; Fernández-Fernández et al., 2020). As expected, *klotho* gene downregulation was found in FA-induced AKI of young mice and in control and FA-AKI old kidneys (Figure 9A). Interestingly, kidney *klotho* mRNA levels were far lower in old FA-injected mice that in young FA-injected mice (Figure 9A), suggesting that *klotho* is a key target gene in AKI in the elderly. PGC-1α is a master regulator of mitochondrial biogenesis with anti-inflammatory and protective functions (Fontecha-Barriuso et al., 2019; Fontecha-barriuso et al., 2020). There was no change in kidney PGC-1α expression at gene level (named *Ppargc1a*) in young FA-injected mice at the lower than usual FA dose used, whereas decreased levels were found in control old kidney and a further downregulation in old FA kidneys (Figure 9B).

## Discussion

**FIGURE 10:**
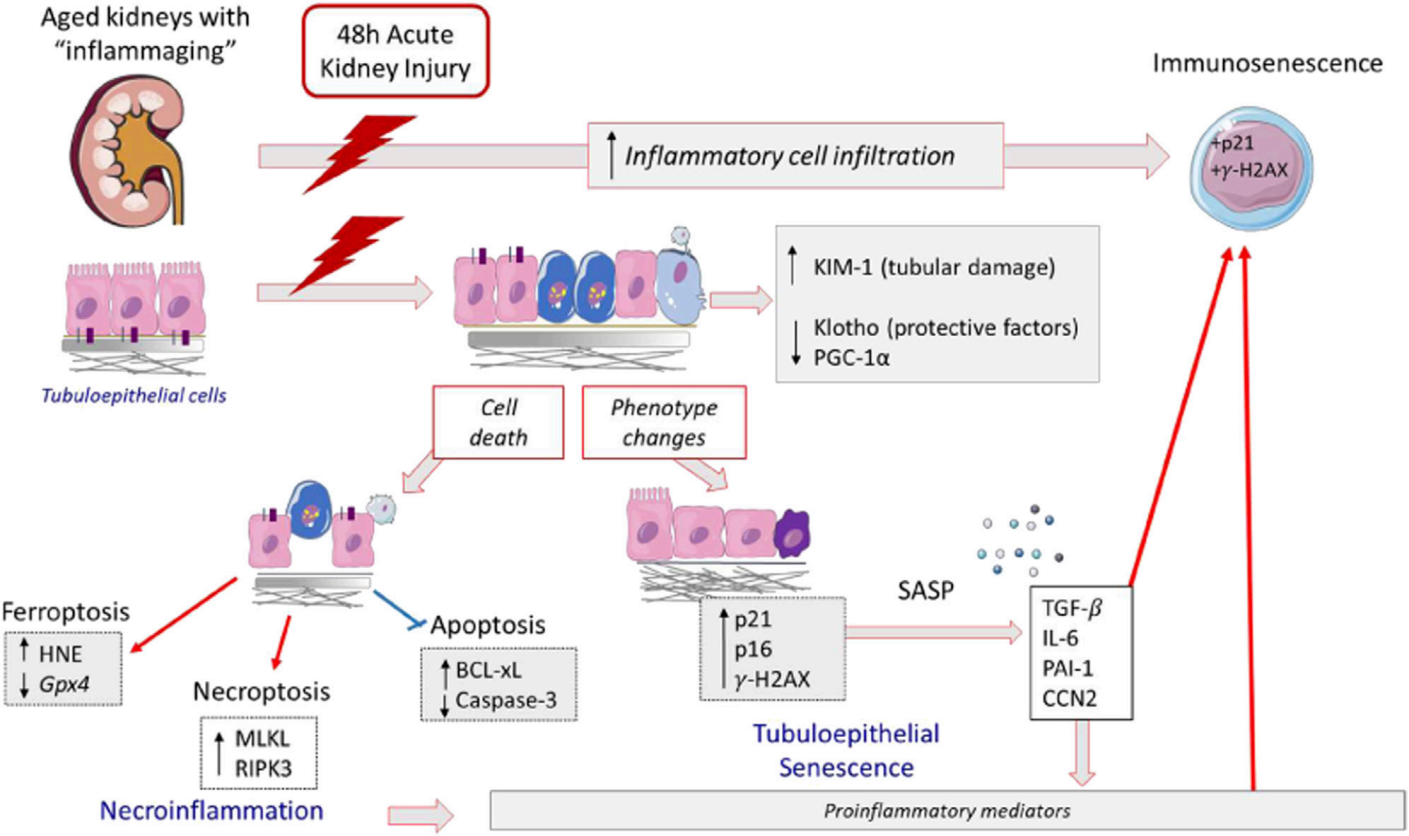
Proposed mechanisms involved in aging-related FA-AKI increased susceptibility. In response to FA injury, aging kidneys present an increase of KIM-1 expression, indicator of tubular damage, lower levels of nephroprotective factors and immunosenescent infiltrating cells. The tubular cell damage can be lethal; in FA-aging kidneys there is an activation of inflammatory forms of cell death, such as necroptosis and ferroptosis, as well as an inhibition of apoptosis. In aging kidneys, injured tubular cells change their phenotype to a proinflammatory and senescent one, being IL-6 one of the most upregulated cytokines. These cellular and molecular changes may partially underlie the age-related increased susceptibility to developing more severe AKI in response to FA.

The studies done in the murine model of AKI by exposure to the toxic compound FA revealed increased acute tubular damage in aging mice. Similarly, more severe drug-related AKI effects in elderly subjects have been described in humans (Metz-Kurschel et al., 1990; Khan et al., 2017), supporting the relevance of this experimental model to explore kidney disease (Miyauchi, 1991; Fan et al., 2017; Montgomery et al., 2017). The characterization of acute tubular damage at molecular level reveals an exacerbation of the tubular injury marker KIM-1 in old mice. The observations regarding the mechanisms triggered by AKI point to an age-related magnification of several proinflammatory-related processes, including gene overexpression of some proinflammatory factors (*Lcn-2*, *Cxcl1*, and *Il-6*), overactivation of inflammatory-cell-death pathways such as necroptosis, and amplification of cellular senescence including immunosenescence (Figure 10). Moreover, our experimental data, showing an exacerbation of renal damage in 12-months-old mice associated with the loss of renal protective factors, support the idea that age-associated susceptibility to AKI may start earlier than previously thought.

After an ischemic or nephrotoxic AKI insult, a wide range of pathophysiological events occur, including changes in tubular cell phenotypes, such as loss of physical cell–cell interactions and partial epithelial-to-mesenchymal transition (EMT) (Ruiz-Ortega et al., 2020), or even tubular cell death mediated by apoptosis and prominent programmed and unprogrammed necrosis (Martin-Sanchez et al., 2018b; Martin-Sanchez et al., 2018a). The proximal tubular cell is an important target of AKI (Chevalier, 2016), as we have confirmed in the FA model by *de novo* expression of the tubular damage biomarker KIM-1 in these cells. Some reports have found a lack of difference in initial severity of IRI, as described by no changes in tubular injury score, between aged and young mice after 1 day post IRI (Sato et al., 2016; Kim et al., 2019), whereas in our model of low-dose of FA-induced AKI, we found an exacerbated increase in KIM-1 positive tubular cells in old mice. Accordingly, in other murine AKI models induced by kidney exposure to toxic compounds, such as heme proteins (Nath et al., 2013), cisplatin (Wen et al., 2015) or bacteria inoculation (Maddens et al., 2012), a significant tubular damage in the acute phase was also described in old mice, showing age-related predisposition of tubular injury in response to toxic-induced damage.

Injured tubular cells are an important source of proinflammatory cytokines and chemokines, which contribute to the amplification of the inflammatory response (Liu et al., 2018). In this sense, FA-injected old murine kidneys presented a synergistic upregulation of proinflammatory genes, such as *Lcn-2*, *Cxcl1*, and *Il-6*, that codify cytokines and chemokines involved in the recruitment of infiltrating immune cells in the kidney. Previous experimental studies have also investigated the inflammatory response in the initial phase of AKI in old mice. In the IRI model, the number of macrophages significantly increased after 1 day in both young and aged mice (Kim et al., 2019), as found in other models of toxic exposure (Nath et al., 2013; Wen et al., 2015). These data clearly indicate that the combination of advanced age and exposure to toxics or ischemia induces an exacerbated innate inflammatory response in the injured kidney at this acute time point and suggest an increased susceptibility of the elderly to AKI. In addition, in the IRI model, exacerbation of immune response and changes in macrophage phenotypes is involved in the AKI-to-CKD transition (Kim et al., 2019).

Many evidences in humans indicate that the elderly exhibit low-grade systemic chronic inflammation even in healthy conditions (Goronzy and Weyand, 2013; Franceschi and Campisi, 2014). Moreover, aging-related dysregulation of several innate and acquired immune responses have been described (Goronzy and Weyand, 2013; Montecino-Rodriguez et al., 2013; Franceschi and Campisi, 2014), and in human kidney transplant patients, aged donor kidneys were observed to attract more infiltrating inflammatory cells than young ones (Øien et al., 2007). Inflammaging of the kidney has also been demonstrated by a microarray analysis of human samples (Rodwell et al., 2004). However, no significant renal changes were found at gene level for proinflammatory factors in healthy old (12 month) mice. Outstandingly, a magnification of the FA-AKI-induced pro-inflammatory response was observed in aging mice, which could be either a cause or a consequence of increased tubular damage. Among the proinflammatory mediators potentially involved in AKI exacerbation, IL-6 has special relevance. We have found that *Il-6* gene expression was synergistically upregulated in FA-AKI in old mice, as previously described in a model of hemoglobin-induced AKI (Nath et al., 2013). Since IL-6 is a proinflammatory cytokine and a SASP component, targeting IL-6 or its downstream signaling could be an interesting therapeutic option in AKI in the elderly.

Tubular cell death is a feature of AKI and both apoptosis and regulated necrosis pathways are activated during FA-AKI (Sanz et al., 2008; Martin-Sanchez et al., 2017; Martin-Sanchez et al.,2018a). Caspase 3 activation is a central event in apoptosis (Justo et al., 2006; Linkermann et al., 2013; Martin-Sanchez et al., 2018a). In the present study, FA-AKI was associated with caspase 3 activation in young but not in old mice. In cultured tubular epithelial cells, inhibition of caspases is known to switch the mode of cell death induced by inflammatory cytokines from apoptosis to necrosis pathways (Justo et al., 2006; Martin-Sanchez et al., 2018a). In accordance with the latter, lack of caspase 3 activation in old FA-AKI mice was associated with evidence of involvement of the necroptosis pathway, i.e., RIPK3 and MLKL upregulation. This is a key difference to point out between young and old FA-AKI mice in our study, since apoptosis is a non-inflammatory form of cell death while necroptosis promotes inflammation (Martin-Sanchez et al., 2018b). In addition, ferroptosis is also overactivated in old FA-AKI, as shown by increased lipid peroxidation. Treatment with Ferrostatin-1, a ferroptosis inhibitor, prevented the inflammatory response and the expression of necroptotic proteins in FA-injected mice (Martin-Sanchez et al., 2017; Martin-Sanchez et al., 2018a), showing that this form of cell death is also related to inflammation. The observed downmodulation of apoptosis in old FA mice is also in line with the induction of a senescent phenotype of tubular cells in injured kidneys, since senescent cells are characteristically protected from apoptosis (Knoppert et al., 2019). These results are supported by the increased baseline and post-FA-induced AKI expression of the antiapoptotic protein BCL-xL showed in old, but not in young mice. In summary, our findings indicate an aging-related change in cell death mechanisms linked to increased tubular injury, characterized by an activation of proinflammatory cell death pathways (necroptosis and ferroptosis) and suppression of non-inflammatory cell death pathways (apoptosis) (Figure 10).

As mentioned above, regulated necrosis can also contribute to age-related amplification of AKI-induced renal inflammatory response. Thereby, DAMPs released by necrotic cells can produce innate immunity cell-derived cytokines by the activation of identical pattern recognition receptors, such as Toll-like receptors expressed on tissue-resident or infiltrating immune cells (Ujiie, 1989; Wu et al., 2007; Kurts et al., 2013). DAMP-associated inflammation is one of the earliest processes following AKI and contributes to an amplification of the loop of cell death/inflammation (Mulay et al., 2016b). Among the immune cells, some studies have demonstrated that macrophages actively participate in necroptosis (Linkermann et al., 2014; Mulay et al., 2016a). In this sense, in the model of IRI, the gene deletion of RIPK3 or MLKL reduced macrophage infiltration and NLRP3 inflammasome activation (Chen et al., 2018). Our results demonstrated that the number of infiltrating macrophages was significantly higher in old FA-injected kidneys associated with an overexpression of the necroptosis components RIPK3 or MLKL, supporting an exacerbation of necroptosis-macrophage inflammatory pathway in aging AKI mice.

Apart from kidney pathologies, necroptosis-mediated inflammation plays an important role in a variety of age-related diseases such as Alzheimer’s disease, Parkinson’s disease, and atherosclerosis (Royce et al., 2019). Some studies have found an association of age-related increase in DAMPs circulating levels, such as mitochondrial DNA or high mobility group protein B1 (Davalos et al., 2013; Pinti et al., 2014), with circulating proinflammatory cytokines (TNF-α, IL-6) in humans, suggesting that DAMPs might play a role in low-grade systemic chronic inflammation described in the elderly (Goronzy and Weyand, 2013; Franceschi and Campisi, 2014). In the same way, some experimental data support a relation between necroptosis and inflammaging. For example, accelerated aging Cu/Zn superoxide dismutase (Sod1) deficient mice that exhibit increased levels of circulating proinflammatory cytokines (Zhang et al., 2013; Deepa et al., 2019) had elevated MLKL protein and gene expression in adipose tissue at 9 months compared with age-matched wild type mice (Royce et al., 2019). Although we have found increased inflammatory cell infiltration in the old mice kidneys, the evaluation of key components of the necroptosis pathway, such as RIPK3 or MLKL, in 1-year old C57BL/6 mice showed no changes at gene and protein levels in healthy kidneys compared to young ones, suggesting that there is no age-related activation of necroptosis in our experimental conditions.

Cellular senescence may occur as a result of cell-cycle arrest due to increased expression of cyclin kinase inhibitors (Knoppert et al., 2019). Previous studies in different AKI models have described a rapid upregulation of p21cip1 expression in the early phase of AKI (Megyesi et al., 1998; Yu et al., 2005; Hodeify et al., 2011). Accordingly, we found increased expression of p21cip1 and p16ink4a in FA-AKI mice. Some studies have proposed that p21cip1 prevents DNA-damaged cells from entering the cell cycle by directly inhibiting CDK2 activity (Yu et al., 2005), thus avoiding cell death by necrosis or apoptosis (Megyesi et al., 1998). Indeed, p21cip1 knockout mice showed increased susceptibility to AKI mediated by ischemia or nephrotoxins (Megyesi et al., 1998; Megyesi et al., 2001; Nishioka et al., 2014). In contrast, the model of renal ablation in p21cip1 knockout mice presented diminished cell-cycle arrest, amelioration of renal dysfunction and lower interstitial fibrosis (Megyesi et al., 1999). On the other hand, renal p21cip1 is essential for the beneficial effects of renal ischemic preconditioning (Nishioka et al., 2014). Moreover, distinct types and severity of kidney injury can behave differently regarding cell-cycle arrest (Yang et al., 2010). Therefore, the functional consequences of p21cip1 expression are cell and disease context specific. In the present study, renal p21cip1 mRNA expression and tubular p21cip1 nuclear staining were significantly higher in old FA-induced AKI than in young mice. Furthermore, the DNA damage response marker γH2AX was also significantly activated in old AKI mice, showing mainly nuclear positive staining in tubular cells. Similarly, activation of prolonged cell-cycle arrest have also been reported in other experimental AKI models, but in this case, linked to fibrosis (Yang et al., 2010). In IRI-AKI mice, treatment with a p53 inhibitor has demonstrated the importance of G1 cell-cycle arrest in the progression of fibrosis (Lim et al., 2018). Another mechanism involved in senescence-mediated renal damage is related to the induction of SASP in injured tubular cells (Acosta et al., 2013). Here, we observed that in FA-induced AKI there was a significant increase in SASP gene expression (including *Tgfβ1, Ctgf/Ccn2*, *Il6*, and *Serpine-1*) in old mouse kidneys. Taken together, this data suggests that there is a magnification of the senescence phenotype in aged AKI mice (Figure 10). Interestingly, our results showed that in old murine injured kidneys, also some infiltrating immune cells were p21cip1 or γH2AX positive, suggesting molecular senescence in the immune cells in the aging kidney may be involved in the aggravated AKI response to FA in old mice (Figure 10). Although the exact cause of inflammaging is not known, cellular senescence (Campisi and D’Adda Di Fagagna, 2007) and immune senescence (Franceschi et al., 2000; McElhaney and Effros, 2009) have been proposed to play a key role in this process.

Finally, yet another remarkable finding was the reduced expression of nephroprotective factors Klotho and PGC-1α and their dramatic further downregulation induced by AKI in old mice. Klotho is normally expressed and secreted by tubular cells and has anti-aging, anti-inflammatory and anti-fibrotic properties (Kuro-o et al., 1997; Kurosu et al., 2005; Sanchez-Niño et al., 2013). Klotho downregulation can be both a consequence and driver of inflammaging in kidney disease (Moreno et al., 2011; Izquierdo et al., 2012; Fernandez-Fernandez et al., 2018; Sanchez-Niño et al., 2019; Fernández-Fernández et al., 2020). For example, Klotho protects endothelial cells from senescence (Carracedo et al., 2012). PGC-1α is the master regulator of mitochondrial biogenesis and PGC-1α deficiency is known to promote spontaneous kidney inflammation and to increase the severity of AKI (Fontecha-Barriuso et al., 2019; Fontecha-barriuso et al., 2020). Thus, the loss of the nephroprotective factors Klotho and PCG-1α due to ageing could contribute to an increased inflammatory and fibrotic response to FA-AKI.

In conclusion, our data indicate that aging kidneys lose local nephroprotective factors and reveal a switch to a proinflammatory cell death (necroptosis and ferroptosis) instead of apoptosis (Figure 10), associated to a synergistic upregulation of several proinflammatory (*Lcn-2* and *Cxcl1*) and SASP mediators, such as IL-6. Moreover, these changes may partially underlie the age-related increased susceptibility to developing more severe AKI in response to toxic compounds, as clearly showed by a dramatic increase of KIM-1 expressing tubular cells (Figure 10). Another characteristic of severe AKI in aging kidneys includes the induction of cellular senescence in intrinsic renal cells and inflammatory cells. These features could interfere with the resolution of acute injury and favor the AKI-to-CKD transition. All these data point out the relevance of investigating the effects of senolytic drugs on cell-death pathways involved in AKI. Better understanding of inflammaging and immunosenescence could contribute to identifying prevention and/or intervention points to mitigate the structural and functional impairment of the kidneys in elderly people. Given the increasing frequency of AKI in the elderly, this information may help to come up with age-specific interventions to prevent or treat kidney injury in this age group.

## Data Availability

The original contributions presented in the study are included in the article/Supplementary Material, further inquiries can be directed to the corresponding author/s.
